# A Labor Division Artificial Gorilla Troops Algorithm for Engineering Optimization

**DOI:** 10.3390/biomimetics10030127

**Published:** 2025-02-20

**Authors:** Chenhuizi Liu, Bowen Wu, Liangkuan Zhu

**Affiliations:** 1 School of Mechanical and Electrical Engineering, Northeast Forestry University, Harbin 150040, China; lchz@nefu.edu.cn; 2 School of Artificial Intelligence and Automation, Huazhong University of Science and Technology, Wuhan 430074, China; bwwu@hust.edu.cn

**Keywords:** labor division, swarm intelligence, bio-inspired computation, mechanical design, image segmentation, engineering optimization

## Abstract

The Artificial Gorilla Troops Optimizer (GTO) has emerged as an efficient metaheuristic technique for solving complex optimization problems. However, the conventional GTO algorithm has a critical limitation: all individuals, regardless of their roles, utilize identical search equations and perform exploration and exploitation sequentially. This uniform approach neglects the potential benefits of labor division, consequently restricting the algorithm’s performance. To address this limitation, we propose an enhanced Labor Division Gorilla Troops Optimizer (LDGTO), which incorporates natural mechanisms of labor division and outcome allocation. In the labor division phase, a stimulus-response model is designed to differentiate exploration and exploitation tasks, enabling gorilla individuals to adaptively adjust their search equations based on environmental changes. In the outcome allocation phase, three behavioral development modes—self-enhancement, competence maintenance, and elimination—are implemented, corresponding to three developmental stages: elite, average, and underperforming individuals. The performance of LDGTO is rigorously evaluated through three benchmark test suites, comprising 12 unimodal, 25 multimodal, and 10 combinatorial functions, as well as two real-world engineering applications, including four-bar transplanter mechanism design and color image segmentation. Experimental results demonstrate that LDGTO consistently outperforms three variants of GTO and seven state-of-the-art metaheuristic algorithms in most test cases.

## 1. Introduction

Engineering optimization aims to identify the best possible solutions (e.g., maximum efficiency, minimum cost) within high-dimensional search spaces governed by physical, economic, or operational constraints. For instance, designing a fuel-efficient aircraft wing requires balancing aerodynamic performance with structural weight limitations. The real world contains countless engineering optimization problems. Mechanical design, pattern recognition, path planning, and other practical engineering can be extracted as optimization models [1]. As the no free lunch theorem proposed, a foundational principle in optimization theory asserts that no single algorithm can universally outperform others across all problem types. For example, an algorithm excelling in smooth unimodal landscapes may fail in rugged multimodal terrains. Thus, developing customized optimizers for engineering arouses intense scholarly interest.

Traditional exact solvers, such as integer programming algorithms, can achieve optimal solutions to small-scale data problems at the theoretical level. However, it is difficult to apply the exact solvers to practical optimization problems due to their unaffordable complexity in time and space. For medium- or large-scale problems, it is difficult to find an optimal solution in a limited time, which requires a compromise and trade-off between solution refinement and computing time. For large-scale problems, it is only necessary to find a suboptimal or satisfactory solution in a short period coupled with the improvement of computer performance. Heuristic algorithms—rule-based methods inspired by natural phenomena (e.g., bird flocking, ant foraging)—have emerged as practical alternatives. Unlike traditional exact methods requiring rigorous mathematical derivations, heuristics prioritize “good-enough” solutions through iterative trial-and-error processes.

A swarm intelligence algorithm is a heuristic optimization algorithm that simulates the behavior of social insects or animals [2]. Individuals in the algorithm can solve complex problems only through simple behavior rules. Compared with traditional optimization methods, swarm intelligence algorithm has the characteristics of being independent of initial values, independent of gradient information, low in function requirements and good in solving performance [3]. The common swarm intelligence algorithms are the ant colony algorithm, particle swarm algorithm, artificial bee colony algorithm, etc. These algorithms have attracted the attention of many scholars and have been widely used in many research fields [4].

The Gorilla Troops Optimizer (GTO) [5] mimics the hierarchical social structure of wild gorilla groups. In GTO, “silverback” leaders guide collective decision-making, while subordinate members explore resource-rich regions through coordinated movements—a metaphor for balancing global exploration and local exploitation in optimization. It offers advantages such as robustness, flexibility and simplicity. It is, therefore, widely used in many areas. Ref. [6] used GTO to minimize the integral time squared error of a fine-tuned power system stabilizer and found that the GTO algorithm has a faster convergence rate and better avoidance of local optima. Ref. [7] proposed a combined method of GTO and artificial neural networks (ANN) for the problem of structural damage diagnosis in recognizing simple supported girder bridges. The proposed method provides higher accuracy and precision compared to using ANN alone. Ref. [8] deployed GTO in a flood scenario, combining a nonlinear muskingen model with a new lateral flow equation to create a hybrid muskingen model with 12 decision variables. Ref. [9] introduced an innovative deep learning-based method for tomato leaf disease (TLD) classification. GTO is used to optimize two variants of deep convolutional neural networks that outperform many recent classification methods. In addition, the literature [10] provides a detailed survey of various engineering cases of GTOs.

However, despite the number of GTO success stories, researchers recognize that GTO has several minor drawbacks, including the absence of self-learning, falling in local minima regions, et al. [11]. To the best of our knowledge, there are currently no studies to analyze and improve the GTO from the perspective of the biological division of labor. Additional related work is presented in Section 2.

The division of labor is a fundamental property of natural biological groups. Labor division provides an efficient, flexible and robust problem-solving framework for AI algorithms by modeling the collaboration patterns of natural systems. Its wide range of applications is used in the fields of swarm intelligence, multi-agent systems, and distributed computing. Ref. [12] proposed a lotus-inspired autonomous task allocation method for multi-UAV missions, improving throughput and task completion time while maintaining scalability, sustainability, and linear running time, benchmarked against four established paradigms. Ref. [13] proposed an ant colony-inspired approach for automated cell tracking using multi-Bernoulli random finite sets, where scouts generate candidates, and worker ants gather heuristic information to estimate cell states, outperforming previous methods in simulations and real image sequences. Biological division of labor hinges on two principles: (1) individual specialization (distinct roles for members) and (2) role plasticity (dynamic task-switching based on environmental cues). For instance, honeybees may transition from nest-building to foraging as floral resources dwindle. Individual specialization means that individuals in a group of organisms perform different tasks and roles. Role plasticity refers to the fact that an individual’s tasks change in response to changes in internal and external conditions. In the initial GTO, all individuals used the same search equation, indicating that individuals are relatively unspecialized. In addition, all individuals in the GTO perform exploration and exploitation searches sequentially in order. This suggests that role plasticity is less malleable when responding to changes in the search environment. Therefore, the labor division in the original GTO is not distinct.

In this work, we introduce the principle of division of labor into the GTO for managing the trade-off between exploration and exploitation. Specifically, the hunting of gorillas in nature involves two processes: the allocation of tasks during the hunt and the allocation of results at the end. As a result, the gorilla’s role division and behavioral development responded adaptively to changes in the search environment, resulting in better search performance of the algorithm in the face of multiple engineering optimization problems. The main contributions of this paper can be summarized as follows:

- We propose a variant of GTO based on the division of labor, which, to the best of our knowledge, is the first study to introduce this natural principle in GTO;
- We delineate the two stages of gorilla foraging. In the division of the labor stage, we design a stimulus-response model for adaptive tuning exploration and exploitation. In the outcome allocation stage, ordinary and elite individuals are designed with search equations for targeted functions;
- We validate our method on three sets of widely used benchmark functions (including 47 test functions) and two real-world engineering cases (four-bar transplanter mechanism design and color image segmentation). The results show that LDGTO has excellent performance for the above engineering optimization problems.

The remaining sections of this paper are arranged as follows: Section 2 is the related work. Section 3 presents the basic optimizer GTO. Section 4 presents our proposed LDGTO algorithm. Section 5 introduces the numerical experiments and analysis. Section 6 illustrates the application in color image segmentation. Section 7 gives the application in a four-bar transplanter mechanism design. Section 8 puts forward the conclusion.

## 2. Related Work

The properties of GTO have attracted the attention of many researchers in recent years. However, the conventional GTO algorithm has a critical limitation: all individuals, regardless of their roles, utilize identical search equations and perform exploration and exploitation sequentially. This uniform approach consequently restricts the algorithm’s performance. To address this limitation, numerous GTO variants have been proposed that efficiently enhance the performance of GTO, as discussed below:

In the field of algorithm discretization, Piri et al. [14] proposed a Discrete Gorilla Troop Optimization (DAGTO) algorithm specifically designed for feature selection (FS). They developed four variants of DAGTO, each tailored to specific health criteria to meet the demands of FS. In addition, they incorporated an information-theoretic-based initialization method to accelerate the convergence speed of the algorithm. To validate its effectiveness, the algorithm was applied to datasets related to COVID-19 patients, yielding positive results. El Houd et al. [15] developed a novel framework leveraging swarm intelligence algorithms to optimize hybrid assembly lines in the automotive industry. They designed two discrete versions of the Whale Optimization Algorithm (VNS-DWOA) and Gorilla Troop Optimizer (DGTO) for assembly line balancing. In practical applications, VNS-DWOA significantly improved cycle time, with enhancements ranging from 7% to 20% compared to expert solutions.

Several improved versions of the GTO algorithm have incorporated chaotic mappings to enhance search efficiency. Chaotic mapping is a mathematical tool used to analyze nonlinear dynamic systems and helps optimize the performance of algorithms. Sayed and Hassanien [16] introduced the Chaotic Gorilla Troop Optimizer (CGTO), which addresses common local optima and premature convergence issues in GTO by integrating chaotic mapping. The study evaluated CGTO’s performance in global optimization and multi-threshold tasks, employing three different chaotic mappings: circular, Gaussian, and tent chaotic maps. Ganguli [17] introduced a chaotic-based Gorilla Troop Optimizer (CBGTO). This version adjusts the tuning parameters and random variables of GTO using one-dimensional chaotic mappings. To assess CBGTO’s performance, he employed a five-phase induction motor model and designed an intelligent PID controller. Statistical analysis, especially the calculation of the time-weighted absolute error (ITAE), demonstrated the significant advantage of CBGTO in control systems, particularly with a 50-horsepower induction motor. Chaudhary and Ganguli [18] refined the GTO algorithm by proposing a chaotic variant, CGTO. This method modifies the position update formula using one-dimensional mappings from ten chaotic maps. The approach exhibited excellent performance in both unimodal and multimodal benchmark functions and was successfully applied to optimize a 50-horsepower induction motor model. Experimental results indicated that CGTO outperforms traditional methods and modern optimization techniques in terms of convergence speed and accuracy.

Additionally, researchers have enhanced the performance of GTO by incorporating opposition-based learning techniques. This integration aims to improve the algorithm’s developmental capability and convergence speed, optimizing the balance between exploration and exploitation. Si et al. [19] utilized GTO and its improved version, GTORBL, to optimize MRI segmentation for breast cancer. The study demonstrated that GTORBL outperformed in terms of segmentation accuracy, sensitivity, and dice similarity coefficient. Vashishtha et al. [20] proposed an enhanced gorilla troop optimization algorithm, which combines opposition-based learning and quantum gate rotation for optimizing hyperparameters in convolutional neural networks. The method achieved a classification accuracy of 98.95% in worm gearbox fault diagnosis, with a standard deviation of 0.2145, showing stronger performance compared to other methods. Alrayes et al. [21] introduced an innovative deep learning-supported cybersecurity threat detection model, which integrates an enhanced gorilla troop optimizer and opposition-based learning-based pinhole imaging strategy to tackle security challenges in IoT cloud networks. The model was evaluated on malignant and benign datasets, achieving an accuracy of 99.47%, demonstrating its superior performance.

Although the aforementioned successful cases have improved GTO from various perspectives, and these algorithms have demonstrated excellent performance in addressing problems within their respective domains, several limitations persist in these GTO variants. These include a lack of self-learning ability, susceptibility to local minima, and weak generalization ability for different problem instances. Biological populations in nature exhibit high adaptability through division of labor. To the best of our knowledge, there has been no research that analyzes and improves GTO from the perspective of biological division of labor.

## 3. Basic Optimizer

The main inspiration of GTO lies in the gorilla troops’ tendency to a communal life among gorillas and continues to live under the leader silverback, which provides it prospective characteristics of well optimization property. Here, we make a brief description of its mechanisms.

Exploration phase: In the GTO algorithm, each gorilla is seen as a candidate solution, and the best solution is considered a silverback gorilla. In the exploration phase, a gorilla could migrate to an unknown location, towards a known location, or approach other gorillas:

(1) $$GX(t+1)=\left\{\begin{array}{l} (UB-LB)\times r_{1}+LB,\;\;\;\;\;\;\;\;\;\;\;\;\;\;\;\;\;rand<p\\ (r_{2}-C)\times X_{r}(t)+L\times H,\;\;\;\;\;\;\;\;\;\;\;\;\;rand\geq 0.5\\ X(t)-L\times (L+r_{3})\times (X(t)-GX_{r}(t)),\;rand<0.5 \end{array}\right.$$

where GX(t+1) represents the candidate position in t+1 iteration; X(t) is the gorilla position; $r_{1},\;r_{2},\;r_{3}$, and rand are random values ranging from 0 to 1; p is a preset parameter ranging from 0 to 1; UB and LB represent the bounds of variables; $X_{r}$ and $GX_{r}$ are randomly selected gorillas; C, L, and H are process parameters whose calculation processes described in detail in [10].

Exploitation phase: Two behaviors of following the silverback and competition for adult females are employed in this phase. On the one hand, the silverback leads the group towards the food source. On the other hand, the silverback may be substituted by stronger gorillas. These mechanisms are illustrated, respectively, as:

(2) $$GX(t+1)=L\times M\times (X(t)-X_{silverback})+X(t)$$

(3) $$GX(t+1)=X_{silverback}-(X_{silverback}\times Q-X(t)\times Q)\times A$$

where $X_{silverback}$ is the best solution; L, Q, M and A are process parameters whose calculation processes can be found in [10], *M* is a position-weighted calculation for all individuals. At the end of the Exploration and Exploitation phase, the fitness of all GX solutions will be calculated and compared with the current optimal. If any GX solution is betted, then the silverback will be substituted.

## 4. Labor Division Artificial Gorilla Troops Algorithm

### 4.1. Task Allocation Phase

Biological communities effectively adapt to environmental changes through labor division [22], of which the stimulus-response division of the labor mechanism is an important manifestation. Under this mechanism, individuals decide whether to participate in a task based on the urgency of the external task. The urgency of the task can be regarded as the intensity of the environmental stimulus, and each individual has a specific response threshold. When the intensity of the environmental stimulus exceeds this threshold, the individual is more inclined to perform the corresponding task. This mechanism can be described by the following mathematical model:

Assuming that there are multiple tasks in the environment, let the number of environmental stimuli for task *i* be denoted as *S_i_*, and the response threshold of an individual to task *i* be $\theta_{i}$, then the probability $\Gamma_{i}$ of an individual performing task *i* is denoted as:

(4) $$\Gamma_{i}=\frac{S_{i}^{2}}{S_{i}^{2}+\theta_{i}^{2}}$$

In the stimulus-response division of labor, the environmental stimulus describes the external drive of the individual to perform the task. The greater the stimulus, the more urgent the task. In the algorithmic search process, exploration and exploitation contradict each other, which can be measured by population diversity. It is generally believed that population diversity is good in algorithmic exploration and poor in algorithmic exploitation. Exploration should be enhanced when population diversity is poor, and exploitation should be enhanced when population diversity is good. Therefore, the stimulus to explore is negatively related to diversity, and the stimulus to exploit is positively related to diversity.

Diversity is the difference between individuals, which reflects the distribution of population, expressed as:

(5) $$d_{i}=\frac{1}{S-1}\sum_{j=1,j\neq i}^{S}\sqrt{\sum_{k=1}^{Dim}{\left(x_{ik}-x_{jk}\right)}^{2}}$$

where *d_i_* denotes the individual *i*, *S* is the number of individuals, *Dim* represents the dimension, *x_ik_* and *x_jk_* represent the positions of the two individuals, respectively.

Normalizing diversity to represent population dispersion:

(6) $$E_{f}=\frac{d_{g}-d_{\min}}{d_{\max}-d_{\min}}$$

where *d_g_* denotes the best individual in the population, *d_max_* and *d_min_* respectively denote the upper and lower bounds of di in the whole population.

The environmental stimuli for the exploration task *S_explore_* and exploitation task *S_exploit_* are defined as:

(7) $$S_{\mathrm{explore}}=1-E_{f}$$

(8) $$S_{\mathrm{exploit}}=E_{f}$$

In the stimulus-response mechanism, response thresholds reflect an individual’s intrinsic tendencies during task performance. When the threshold is small, the individual’s tendency is stronger. Under the group reward and punishment mechanism, thresholds decrease when individuals complete a task and increase when they fail to complete a task [23]. The success rate of an individual in performing a task can be used to measure the threshold value of its corresponding task; the higher the success rate, the lower the threshold value. Therefore, when gorillas perform exploration and exploitation tasks, the threshold of their corresponding task is negatively correlated with the success rate.

The mathematical model of the response threshold is as follows. First, for a given task *i*, let *n_i_* denote the total number of times an individual performs the task *i*, and *n* denote the total number of times an individual performs the task successfully. Then the success rate *r_i_* of performing the task *i* can be calculated as:

(9) $$r_{i}=\frac{n_{i}}{n}$$

Secondly, given the negative relationship between success rate and response threshold. $r_{\mathrm{explore}}$ and $r_{\mathrm{exploit}}$ represent the success rate of exploration and exploitation tasks. The response thresholds for the exploration task $\theta_{\mathrm{explore}}$ and exploitation task $\theta_{\mathrm{exploit}}$ in individuals can be defined as follows:

(10) $$\theta_{\mathrm{explore}}=1-r_{\mathrm{explore}}$$

(11) $$\theta_{\mathrm{exploit}}=1-r_{\mathrm{exploit}}$$

In the end, the likelihood Γ of individuals engaging in exploration or exploitation tasks during the allocation phase is determined as follows:

(12) $$\Gamma_{\mathrm{explore}}=\frac{S_{\mathrm{explore}}^{2}}{S_{\mathrm{explore}}^{2}+\theta_{\mathrm{explore}}^{2}}$$

(13) $$\Gamma_{\mathrm{exploit}}=\frac{S_{\mathrm{exploit}}^{2}}{S_{\mathrm{exploit}}^{2}+\theta_{\mathrm{exploit}}^{2}}$$

### 4.2. Outcome Allocation Phase

During the outcome distribution phase, the “strong to weak” principle directs more resources toward the more capable gorillas at the top, enabling them to sustain or enhance their dominance. In contrast, weaker gorillas are deprived and gradually eliminated, relinquishing their share of limited survival resources. This mechanism fosters the progression and refinement of the population. To mirror this outcome distribution rule, the paper introduces prominent roles to strengthen the powerful and lagging roles to phase out the less capable.

Elite individuals usually have high fitness values. In a multi-peak optimization problem, the higher the fitness value of an individual, the higher the probability that it is close to the global optimal solution. Therefore, further local development should be carried out in the region where these individuals are located to improve the quality of the solution. To this end, the population is first sorted in descending order according to the fitness of the individuals, and then the probability *P_i_* of the individual *i* performing the localized search is set as:

(14) $$P_{i}=rank_{i}/S$$

where *rank_i_* denotes the ranking of individual *i* in the population.

Based on the aforementioned formula, we can see that the fitness-ranked selection method focuses more on retaining elite individuals in the evolutionary process. Especially when facing the minimization problem, lower-ranked individuals are susceptible to the significant influence of higher-ranked elite individuals. This selection method helps to maintain the stability of the algorithm. After the selection process, a Gaussian distribution method is used to perform a localized search for individuals. In the local search, effective exploration of the solution space can be achieved by sampling a small area around the expectation value, thus improving the accuracy and efficiency of the local search. The search equation is of the following form:

(15) $$X_{new}=Gaussian(X,\sigma )$$

where *X_new_* denotes the new individual position obtained by local search and σ is the standard deviation. If a higher quality solution is obtained by local search, the existing solution is replaced.

Individuals exhibiting suboptimal performance, often situated at a considerable distance from either the global optimum or local optima, tend to diminish the population’s search efficiency and elevate the risk of convergence to local optima. However, it is noteworthy that individuals demonstrating poor fitness during the initial stages of evolution may not necessarily remain underperforming in subsequent iterations. Consequently, identifying such individuals as lagging members inherently involves forecasting their future fitness potential. To strike a balance between computational efficiency and the imperative of preserving population diversity [24], this study employs a linear prediction approach to estimate the prospective fitness of individuals. Specifically, the improvement potential of an individual is quantified as follows:

(16) $$I_{i}^{t}=f_{i}^{t-1}-f_{i}^{t}$$

where $I_{i}^{t}$ denotes the improvement of the individual *i* in the *t*-th generation and $f_{i}^{t}$ denotes the adaptation of an individual *i* in the *t*-th generation.

Then, based on the improvement of the solution and the number of remaining iterations, the final fitness of the individual is predicted to be:

(17) $$f_{i}^{t_{\max}}=f_{i}^{t}-(t_{\max}-t)\cdot I_{i}^{t}$$

After obtaining the predicted final fitness of an individual, it is compared to the optimal solution in the current population. If the fitness of an individual is lower than the current optimal solution, it means that the region in which the individual is located is not valuable for further search, and thus, these poorly performing individuals will be reinitialized to start the process of global exploration. Since the population size and resources are generally limited, the reinitialization process can be seen as the good individuals regaining more resources for survival. In the early stages of evolution, poorly performing individuals are usually eliminated less frequently due to greater individual improvements, allowing for deeper exploration of promising areas and more efficient exploitation of those areas. However, as evolution progresses, the population gradually aggregates, and the room for improvement becomes smaller, the role of the elimination mechanism becomes particularly important, which can not only effectively avoid ineffective searching of areas with no potential, but also avoid repeating meaningless searching of areas that have already been explored, to improve the search efficiency, and avoid the waste of resources.

**Figure 1:**
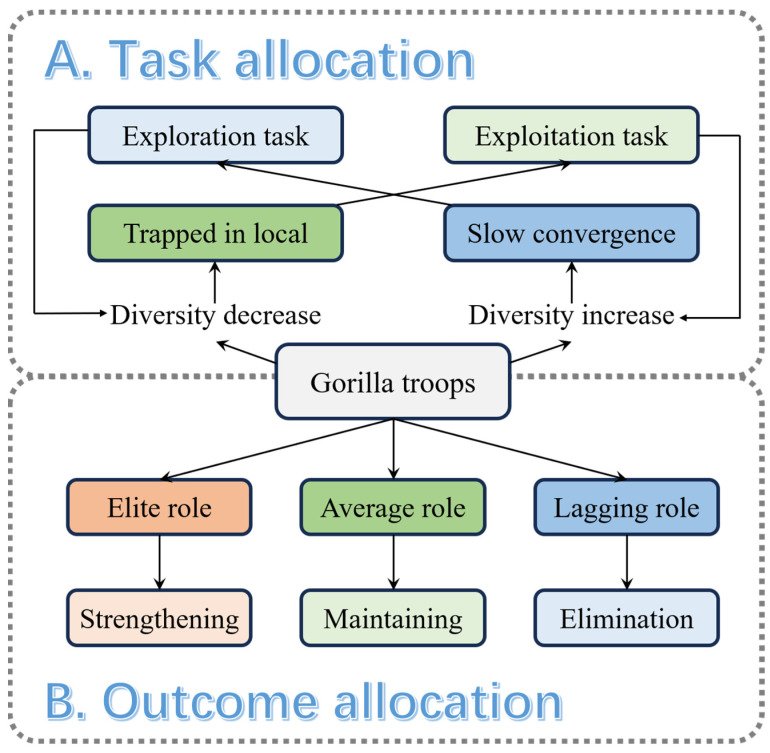
Two-stage labor division mechanism in LDGTO.

Synthesizing the above mechanisms, a schematic is given in Figure 1.

### 4.3. The Framework of the LDGTO

**Figure 2:**
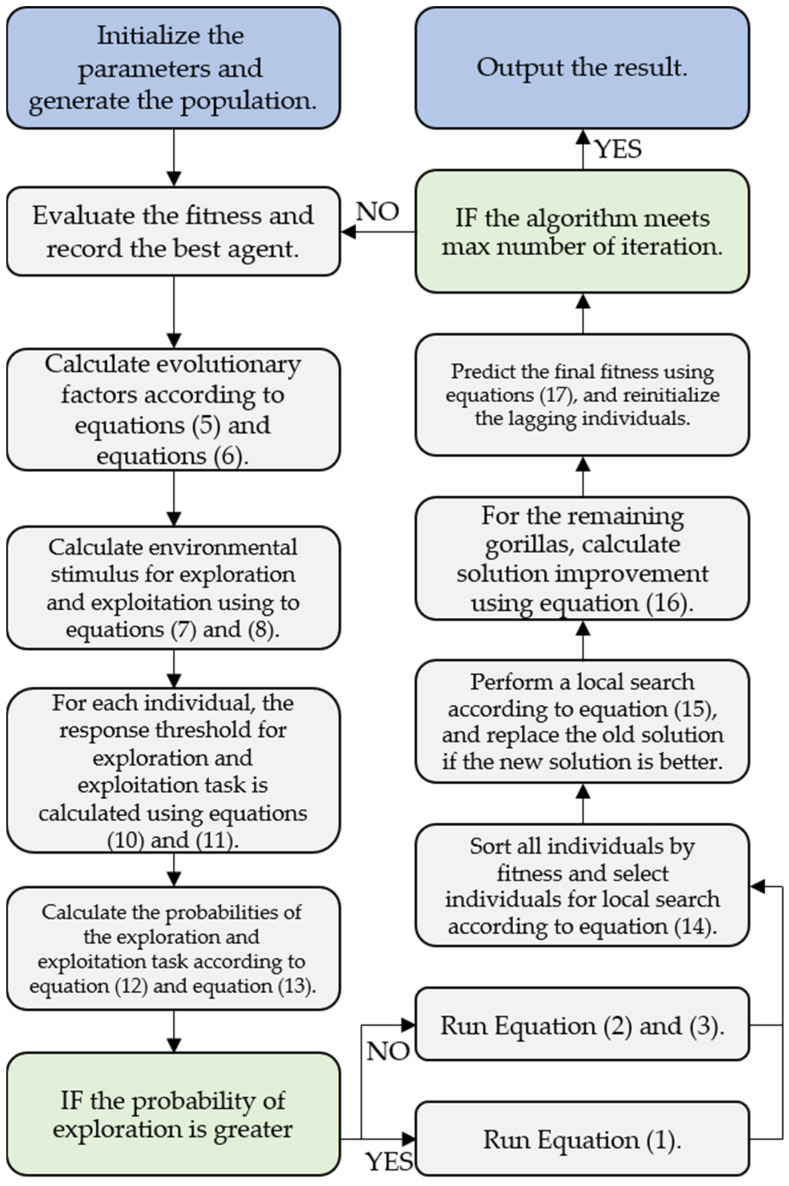
Workflow of the LDGTO algorithm.

To show the algorithm-building process more clearly and concisely, the implementation process of LDGTO is presented in Figure 2.

### 4.4. Convergence Analysis

#### 4.4.1. Basic Assumptions and Definitions

Consider the optimization problem as a minimization problem, where the objective function $f:{\mathbb{R}}^{d}\to \mathbb{R}$ is continuous on a compact set $\chi \subset {\mathbb{R}}^{d}$, and a global optimal solution $x^{*}$ exists with the corresponding fitness value $f^{*}$. The population size of LDGTO is *S*, and the maximum number of iterations is *t_max_*. According to the convergence theorem for stochastic search algorithms by Solis and Wets [25], if the algorithm satisfies the following conditions, it converges to the global optimal solution with probability 1 [26,27,28]:

- Global Coverage: For any solution x∈χ and any iteration *t*, there is a non-zero probability of generating *x*;
- Elite Retention: If the population at any generation contains the global optimal solution $x^{*}$, then $x^{*}$ is retained in all subsequent iterations.

The following sections verify these conditions through the algorithm’s various stages.

#### 4.4.2. Verification of Global Coverage

Global coverage is mainly guaranteed by the exploration phase, re-initialization and labor division mechanisms.

##### Exploration Phase

The candidate solution generation mechanism in the exploration phase is:

$$GX(t+1)=\left\{\begin{array}{l} (UB-LB)\times r_{1}+LB,\;\;\;\;\;\;\;\;\;\;\;\;\;\;\;\;rand<p\\ (r_{2}-C)\times X_{r}(t)+L\times H,\;\;\;\;\;\;\;\;\;\;\;\;\;rand\geq 0.5\\ X(t)-L\times (L+r_{3})\times (X(t)-GX_{r}(t)),\;rand<0.5 \end{array}\right.$$

where $r_{1},r_{2},r_{3},rand\in [0,1]$ are uniformly distributed random numbers and $X_{r}(t)$ and $GX_{r}(t)$ are randomly selected individuals. Since:

- The first branch generates a new solution randomly within the search space via $(UB-LB)\times r_{1}+LB$;
- The second and third branches generate new solutions by perturbing the current solution or known solutions;
- The parameter p∈(0,1) ensures that each branch is executed with a non-zero probability.

Therefore, the exploration phase can cover any x⊂χ with a non-zero probability, satisfying global coverage.

##### Labor Division Mechanism

In Equations (4)–(13), the task allocation probability is determined by environmental stimuli and response thresholds:

$$\Gamma_{\mathrm{explore}}=\frac{S_{\mathrm{explore}}^{2}}{S_{\mathrm{explore}}^{2}+\theta_{\mathrm{explore}}^{2}},\;\Gamma_{\mathrm{exploit}}=\frac{S_{\mathrm{exploit}}^{2}}{S_{\mathrm{exploit}}^{2}+\theta_{\mathrm{exploit}}^{2}}$$

where $S_{\mathrm{explore}}=1-E_{f}$, $S_{\mathrm{exploit}}=E_{f}$, and $E_{f}$ is calculated by normalizing population diversity, Equations (5) and (6). When population diversity is low ($E_{f}\to 0$), $S_{\mathrm{explore}}\to 1$, and the algorithm tends to explore; otherwise, it tends to exploit. This mechanism dynamically adjusts the exploration probability, preventing premature stagnation and further ensuring global coverage.

##### Reinitialization of Lagging Individuals

For individuals with predicted fitness lower than the current optimal (lagging individuals), their positions are reinitialized:

$$f_{i}^{t_{\max}}=f_{i}^{t}-(t_{\max}-t)\cdot I_{i}^{t}$$

Reinitialization is equivalent to randomly generating new solutions within the search space, supplementing global exploration capabilities and preventing the population from becoming trapped in local regions.

#### 4.4.3. Verification of Elite Retention

##### Silverback Retention Mechanism

In each iteration, the silverback gorilla (current best solution $X_{silverback}$) is explicitly retained. If the population at any generation contains $x^{*}$, its fitness $f^{*}$ cannot be surpassed, so $x^{*}$ will always be retained in subsequent iterations.

##### Elite Local Search

Elite individuals are ranked by fitness, and local search is performed with probability $P_{i}=rank_{i}/S$:

$$X_{new}=Gaussian(X,\sigma )$$

where *Gaussian* perturbation samples a small area around the current solution. Since only better solutions are replaced, the local search around elite individuals does not compromise their retention while improving local search accuracy.

#### 4.4.4. Convergence Conclusion

Combining the above analysis, LDGTO satisfies the global coverage and elite retention conditions of the Solis–Wets theorem. Therefore, for optimization problems on continuous compact sets, the algorithm converges to the global optimum with probability 1:

$$\underset{t\to \infty }{\mathrm{Lim}}P(\exists x\in P_{t},f(x)=f^{*})=1.$$

#### 4.4.5. Additional Remarks

- Labor Division Mechanism (Equations (4)–(13)) dynamically balances exploration and exploitation through environmental stimuli and response thresholds, avoiding premature convergence;
- Local Search (Equation (15)) and Reinitialization (Equation (17)) complement each other by enhancing local exploitation and global exploration, respectively;
- Markov Chain Modeling: The iterative process of the algorithm can be modeled as a Markov chain ${\left\{P_{t}\right\}}_{t=0}^{t_{\max}}$, whose state transition kernel satisfies the convergence conditions [25].

## 5. Numerical Experiments and Analysis

In this section, numerical function sets are introduced to measure the efficiency of the proposed LDGTO. The experiment is divided into two rounds. In the first round, 12 unimodal functions and 25 multimodal functions are selected to compare LDGTO with several GTO variants. In the second round, 10 more complex combinatorial functions from CEC 2017 are employed. LDGTO versus popular other peers and deterministic method.

### 5.1. Experiment Setup

In the first round, two sets of benchmark functions containing 12 unimodal functions and 25 multimodal functions are adopted; see Table 1. These sets are commonly utilized [29]. The unimodal problems are generally employed for evaluating exploitation capability. The multimodal functions are favored to evaluate the exploration ability and the comprehensive nature. The information on the above-tested functions is shown in Table 1 and Table 2.

For comparison, the classic GTO and two GTO variants are used as experimental comparison algorithms, including GTOT [30] and mGTO [31], and the unique parameter settings of each algorithm are consistent with their references. For the common parameters, the number of population *S* = 30, and the maximum number of iterations *t_max_* = 500. The experiment is implemented in the MATLAB R2016b software, and simulations are run on a computer with Intel CPU @2.20 GHz and 12 GB RAM.

### 5.2. Comparison with GTO Variants on Unimodal and Multimodal Functions

The composite measure of a meta-heuristic usually takes the talent of (1) exploiting the prospective area and (2) exploring the entire search space. In this section, a comparative evaluation of LDGTO is made against the prototype with all three aspects considered.

Exploitation capability. In analyzing the results of the unimodal function optimization experiments, we will focus on the metrics of each algorithm’s ability to converge to the global optimal solution during the optimization process, solution accuracy, and stability. The unimodal function is characterized by only one global optimal solution, and the task of the algorithms is to find that optimal solution quickly and accurately while avoiding falling into local solutions with unstable values or large errors.

Table 3 lists the optimization results of the four algorithms (GTOT, mGTO, GTO, and LDGTO) on unimodal functions. Each row represents a test function and contains the final optimization results of the four algorithms on that function. The last row of the table shows how LDGTO compares with the other three algorithms, where:

- LDGTO algorithm outperforms the other algorithms, indicating that LDGTO significantly outperforms the other algorithms on a particular function;
- LDGTO is comparable to the other algorithms, indicating that LDGTO performs similarly to the other algorithms in terms of performance, with a small gap;
- LDGTO Lagging to other algorithms indicates that LDGTO does not perform as well as other algorithms in specific cases.

In Table 3, it is evident that the LDGTO algorithm demonstrates significantly better performance in unimodal problems, as highlighted in the detailed analysis. LDGTO obtains greater grades than GTO peers on most of the 12 problems, to be specific:

- In unimodal functions, the most important goal of the algorithm is to find the globally optimal solution and minimize the computational error. Taking UF1 (1.60 × 10^4^, 6.67 × 10^4^, −2.02 × 10^4^, and 3.25 × 10^−74^) as an example, the results of GTOT and mGTO deviate from each other considerably, while the result of LDGTO algorithm is 3.25 × 10^−74^, which is close to zero, and it can be regarded as an extremely accurate globally optimal solution, which demonstrates its powerful ability in unimodal optimization.
- On UF4 (7.31 × 10^2^, 1.08 × 10^3^, −3.29 × 10^2^, and 1.88 × 10^−59^), the results of GTOT and mGTO deviate from the expected optimal solution, especially mGTO (1.08 × 10^3^), which is far from the expected optimal solution, while the LDGTO algorithm converges to a very small value close to zero, showing its stable numerical performance. For UF6 (1.43 × 10^4^, 1.10 × 10^5^, −8.45 × 10^4^, and 3.89 × 10^−1^), although the LDGTO results in 3.89 × 10^−1^, it still exhibits a relatively small error compared to the other algorithms.
- In some special functions, such as UF7 (−2.75 × 10^2^, −2.75 × 10^2^, −2.75 × 10^2^, and −2.77 × 10^2^), all algorithms give close results with small deviations. This suggests that the function is relatively flat and difficult to optimize further, so there is little difference in performance between the algorithms. However, in function UF10 (−4.71 × 10^2^, 6.20 × 10^2^, 1.23 × 10^3^, and 3.55 × 10^1^), LDGTO’s result of 3.55 × 10^1^ is much better than the other algorithms, demonstrating its ability to avoid large local optimal solutions and find a composite solution.
- In very small value problems such as UF11 (−6.87 × 10^−48^, −4.06 × 10^−47^, 2.34 × 10^−46^, and 1.43 × 10^−63^), the results of GTOT and mGTO have a large deviation, whereas LDGTO shows great numerical accuracy, obtaining a result of 1.43 × 10^−63^, which is much smaller than that of the other algorithms, indicating that it has a significant advantage in dealing with very small value and high accuracy requirement problems with very small values and high accuracy requirements, indicating that it has a significant advantage in dealing with problems with very small values and high accuracy requirements.

In summary, the LDGTO algorithm in unimodal function optimization presents advantages. It demonstrates obvious advantages in most of the tested functions, especially those requiring high accuracy and stability (e.g., UF1, UF4, UF11), and shows strong global optimal solution approximation ability.

Although the LDGTO algorithm demonstrates significant performance advantages in most test cases, it slightly underperforms comparison algorithms such as GTOT, mGTO, and standard GTO in a very small number of unimodal function optimization problems. This includes slightly inferior to GTO at UF5, losing to GTOT, mGTO, and GTO at UF9 and UF12. This phenomenon is not an intrinsic flaw in the algorithm design but is closely related to problem characteristics, the trade-off between algorithmic exploration and local search capabilities, and the domain suitability of the comparison algorithms. In unimodal function optimization, only a single globally optimal solution for the objective function exists, which requires high local search capability (Exploitation) and low global exploration (Exploration) of the algorithm. The core innovation of LDGTO is to enhance the global exploration capability through a dynamic hierarchical strategy to cope with complex multi-peak, high-dimensional, or noise-containing optimization problems. However, in very few low-dimensional unimodal functions, comparative algorithms such as GTOT and mGTO may have an advantage in initial convergence speed due to their purposefully designed local convergence strategies (e.g., parameter adaptive mechanism based on power system tidal current problem in GTOT and improved local search operator in mGTO). Nevertheless, LDGTO still exhibits significantly better or equivalent performance in the vast majority of unimodal functions, and its global exploration capability is irreplaceable in complex optimization problems.

Exploration capability: The challenge of multimodal function optimization is that it contains multiple local optimal solutions. The algorithms not only need to have strong exploration capabilities to avoid falling into local optimal solutions but also need to have the ability to balance exploration and exploitation.

Table 4 gives the results of algorithms for multimodal functions. For a simple visualization, the comparison between the LDGTO algorithm and each comparison algorithm is given in the last row of the table. Where +/=/− indicates that the LDGTO algorithm performs better on + function, comparable on = function, and worse on − function compared to this algorithm. In Table 4, the LDGTO algorithm achieves superior performance in multimodal problems, as can be observed in the specific analysis:

- Taking MF4 (3.41 × 10^10^, 2.63 × 10^11^, −5.57 × 10^11^, and 1.23 × 10^4^) as an example, the results of the GTOT and mGTO algorithms deviate considerably from this function, which may indicate that they do not find sufficiently effective regions during the global search. The result of LDGTO (1.23 × 10^4^), on the other hand, is significantly closer to the more optimal solution, reflecting its stronger exploration capability;
- For functions such as MF12 (−3.47 × 10^−1^, −1.00 × 10^0^, 1.39 × 10^0^, and −1.00 × 10^0^), the results of all the algorithms are close to the optimal value to different degrees, and LDGTO can effectively avoid falling into local optimality. Especially when dealing with very small value problems similar to MF3 and MF13 (6.25 × 10^−21^, −1.39 × 10^−20^, 2.94 × 10^−20^, and 1.70 × 10^−108^), the LDGTO algorithm shows higher adaptability and stability;
- MF9 (1.37 × 10^1^, 2.90 × 10^1^, −1.04 × 10^2^, and 5.96 × 10^−50^) function shows that the LDGTO algorithm can break through the obstacles of local optimal solutions encountered by GTOT and mGTO, and explores a region that is closer to the global optimal solution. Its result (5.96 × 10^−50^) demonstrates its extreme global search capability;
- For functions MF17 and MF18 (−1.96 × 10^2^, −1.96 × 10^2^, −1.96 × 10^2^, −1.96 × 10^2^, and −1.96 × 10^2^), the results of the individual algorithms are very close to each other, suggesting that the optimization of the algorithms in this type of flat multimodal functions is relatively less difficult, and the various algorithms show similar robustness. However, for more complex functions, LDGTO shows more competitiveness.

Through the in-depth analysis of the data in Table 4, the following conclusions can be drawn: the LDGTO algorithm performs well in most of the tested functions, especially in solving complex multimodal functions with multiple locally optimal solutions, and its global search ability and avoidance of locally optimal solutions are much better than GTOT, mGTO, and GTO algorithms.

Analyze scenarios in which the LDGTO does not win. LDGTO only show inferior to GTOT, mGTO, and GTO at MF16. Such multimodal function often be characterized by a low-dimensional, symmetric local optimal distribution, whose global optimal solution is surrounded by dense suboptimal solutions with smooth gradient changes. Such scenarios require very high local search efficiency of the algorithm, and the core innovation of LDGTO—labor division strategy—copes with the complexity of high-dimensional multi-peak problems by enhancing the global exploration capability. In such low-dimensional special cases, the initial convergence speed may be slightly slower due to the hierarchical iterative of resource allocation, leading to slightly slower initial convergence. In contrast, GTOT and mGTO algorithms optimize local search operators (e.g., gradient-following mechanism, elite individual retention) for specific domains (e.g., power systems, feature selection), thus taking advantage of the multi-peak problems with a clear tendency of low-dimensional single-peaking. However, the design goal of LDGTO focuses on high-dimensional, nonlinearly strongly coupled multi-peak scenarios, where its dynamic hierarchical mechanism effectively circumvents dimensional catastrophe and demonstrates significant superiority in the remaining 24 test problems. This trade-off suggests that the innovative power of LDGTO is reflected in its generalizability to complex problems rather than pursuing extreme performance for specific simple cases.

### 5.3. Comparison with Popular Algorithms on Composite Functions

To provide a more thorough validation of the LDGTO algorithm’s effectiveness, we applied it to solve all the problems from the combinatorial functions section of the CEC2017 test set, which included a total of 10 distinct combinatorial functions labeled CF1 through CF10. These problems were designed with a high level of complexity, incorporating a combination of unimodal and multimodal functions. To further increase the difficulty, rotations and offsets were introduced to alter the positions of the optimal solutions, thereby making the functions more intricate and challenging to solve.

In this section, the LDGTO algorithm is evaluated and compared with four well-established algorithms known for their high performance: PSO [32], MFO [33], SCA [34], and GPC [35]. These algorithms were selected due to their popularity and demonstrated effectiveness. For the experiments, each combination function (CF1 to CF10) is tested with a dimensionality of 10 and a maximum of 10,000 evaluations.

As seen in Table 5, among the optimization results for all the combined functions, LDGTO significantly outperforms all the compared algorithms (PSO, MFO, SCA, and GPC) in terms of search accuracy. The mean (Mean) of LDGTO is consistently lower than the other algorithms in all problems, showing its superior performance in the optimization process. For example, in the optimization of CF1 to CF9, the Means of LDGTO are 2.05 × 10^3^, 2.29 × 10^3^, 2.45 × 10^3^, 2.53 × 10^3^, 2.80 × 10^3^, 2.85 × 10^3^, 2.98 × 10^3^, 3.23 × 10^3^, and 3.11 × 10^3^, whereas the Means of the comparison algorithms are generally higher, which shows that LDGTO in these test problems provides higher optimization accuracy.

In addition, the standard deviation (Std) of LDGTO also exhibits smaller values in most problems, indicating that the algorithm has a very high stability. For example, in CF1, the standard deviation of LDGTO is 5.69 × 10^0^, which is much smaller than that of other algorithms, which is 1.22 × 10^3^, which shows its ability to maintain consistent optimization results under different test problems. Compared with other algorithms, the standard deviation of LDGTO is generally much lower, further proving its stability and robustness over multiple runs. In particular, LDGTO performs particularly well on CF10, with a mean value of 1.40 × 10^5^, which is significantly better than the results of algorithms such as PSO (4.56 × 10^5^), MFO (5.21 × 10^5^), and SCA (9.25 × 10^5^), and with a smaller standard deviation (1.20 × 10^5^), demonstrating its excellent performance on the more difficult problems.

Overall, LDGTO shows better performance than the comparison algorithms in the optimization of all combinatorial functions, not only in terms of search accuracy but also in terms of its stability. This indicates that LDGTO can provide more accurate and reliable optimization results when dealing with complex optimization problems, further validating its effectiveness in the labor division.

### 5.4. Comparison with Deterministic Method

Lipschitz global optimization algorithms are deterministic methods that have been fully studied. These methods are usually more complicated than meta-heuristic methods in technology, and their implementation is not easy. They need more memory and higher mathematical preparation to understand and use them. Usually, they have a strong theory to ensure convergence to a global solution and a small number of control parameters so that their users can configure search easily.

Using the MATLAB Toolbox, we compare LDGTO with a very popular Lipschitz global optimization algorithm, DIRECT [36], on the composite function test set of CEC2017. To compare the deterministic algorithm and stochastic algorithm properly, the experimental parameters of the comparison algorithm refer to the literature [37]. The results are reported in Table 6.

In the experiments of multiple composite functions, DIRECT is better than LDGTO in terms of solving quality, especially in CF1–4. We still find that LDGTO can achieve very similar results with the deterministic algorithm in CF5, CF7, CF8, and CF10. In terms of computational efficiency, LDGTO usually outperforms DIRECT. DIRECT obtains the optimal solution through accurate global search, but its calculation cost increases rapidly with the increase of dimensions. Because of its parallel computing characteristics, LDGTO can evaluate more candidate solutions in the same computing time, showing faster convergence speed and higher computing efficiency. This characteristic makes it valuable in practical engineering, especially in the problem of giving the scheme in a limited time.

### 5.5. Parameter Analysis

This section performs a parameter analysis. LDGTO’s primary advantage is its structural separation of exploration (global search) and exploitation (local search), as well as its automatic balance. It introduces only one critical parameter—the local search step size σ in Equation (15). In LDGTO, the values of σ are respectively set to 10^−6^/10^−5^/10^−4^/10^−3^/10^−2^. The experimental results of these five values are listed in Table 7. The last line in the table shows the comparison result of $\sigma =10^{-4}$ with other cases on 10 combinatorial functions. It can be seen that this parameter is not particularly sensitive, and there is no significant performance difference in most functions with different values. But when the value is $\sigma =10^{-4}$, the overall best result can be obtained.

As a summary, the analyses appraise the potential property of our proposed LDGTO algorithm from unimodal, multimodal, and composite functions. It can be observed that the labor division strategy assists the enhanced method in achieving better performance in three aspects than the prototype. In the following section, we further develop LDGTO to tackle two real-world engineering cases (four-bar transplanter mechanism design and color image segmentation).

## 6. LDGTO Handles Color Image Segmentation

Image segmentation plays a crucial role in the computer vision domain, such as face recognition, medical diagnosis, and automatic driving. In comparison to the gray image, the color image provides more abundant information. However, choosing the appropriate threshold for color image segmentation is also more difficult because it involves the combination of three-color spaces.

### 6.1. Problem Setting

In the multi-threshold segmentation problem, assume that the input image $I\in {\mathbb{R}}^{H\times W\times C}$ is an image with three color channels (Red, Green, and Blue), where H and W represent the height and width of the image, and C is the number of color channels, C=3. For each channel c∈{R,G,B}, the pixel intensity values range from [0, *L*−1], where *L* is the maximum intensity value of the pixels. The segmentation of each channel *c* is accomplished by selecting *n* thresholds $t_{c,1},t_{c,2},\text{...},t_{c,n}$ which divide the intensity range into *n* + 1 intervals.

The quality of the segmentation is measured by Kapur entropy. For each channel *c*, its Kapur entropy $H_{c}$ is defined as:

(18) $$H_{c}(t_{c,1},\text{...},t_{c,n})=-\sum_{i=0}^{t_{c,1}}p_{i}^{c}\ln(p_{i}^{c})-\sum_{i=t_{c,1}+1}^{t_{c,2}}p_{i}^{c}\ln(p_{i}^{c})-\cdots -\sum_{i=t_{c,n-1}+1}^{L-1}p_{i}^{c}\ln(p_{i}^{c})$$

here, $p_{i}^{c}$ represents the probability of pixel intensity *i* in channel *c*, which is typically estimated from the image’s pixel distribution. To find the optimal segmentation scheme, the goal is to maximize the total entropy *H*_total_, which is the sum of the Kapur entropies of all three channels:

(19) $$H_{\mathrm{total}}=H_{R}(t_{1,1},\ldots ,t_{1,n})+H_{G}(t_{2,1},\ldots ,t_{2,n})+H_{B}(t_{3,1},\ldots ,t_{3,n})$$

in this formula, *H_R_*, *H_G_*, and *H_B_* represent the Kapur entropies of the red, green, and blue channels, respectively. Our optimization objective is to select the set of thresholds $\left\{t_{c,1},\ldots ,t_{c,n}\right\}$ for each channel that maximizes *H*_total_, i.e.,

(20) $$\{t_{1,1}^{*},\cdots ,t_{1,n}^{*},t_{2,1}^{*},\cdots ,t_{2,n}^{*},t_{3,1}^{*},\cdots ,t_{3,n}^{*}\}=\arg\underset{t_{1,1},\ldots ,t_{1,n},t_{2,1},\ldots ,t_{2,n},t_{3,1},\ldots ,t_{3,n}}{\max}H_{\mathrm{total}}$$

Through this optimization process, we aim to find the optimal set of thresholds that maximizes the information gain of the multi-channel segmentation, thereby achieving a more accurate segmentation result.

### 6.2. Experimental Setup

The experiment is conducted on two groups of bench images: 6 animal images and 5 human images from the BSDS500 dataset. The original images are shown in Table 8. All images are resized to 481 × 321 pixels. Segmentation tests are performed with threshold values set at 2, 3, and 4. The experimental conditions are kept consistent with those used in the previous section. The comparison peers include GTO (2021) [10], POA (2021) [38], WOA (2016) [39], SCA (2016) [34], and MVO (2016) [40].

### 6.3. Performance Criteria

Performance criteria offer a numerical assessment of the thresholded segmented image’s quality. Intra-region uniformity (NU) and inter-region contrast (RC) are essential metrics for evaluating thresholding performance. Their unsupervised nature allows for the assessment of any segmented image, with the added advantage of supporting self-tuning. These metrics relate to uniformity and contrast, which are pivotal in multilevel thresholding techniques.

*NU* is an intra-region uniformity indicator, one of the most intuitive metrics being able to quantify the quality of a thresholding segmentation result. This criterion uses the variance to calculate the homogeneity of a character throughout an area. *NU* can be calculated as follows:

(21) $$NU=1-{\sum_{k=1}^{N_{R}}\sum_{s\in R_{k}}\left[I(s)-\frac{1}{A_{k}}\sum_{s\in R_{k}}I(s)\right]}^{2}=1-\sum_{k=1}^{N_{R}}\sigma_{k}^{2}$$

where $N_{R}$ is the number of regions, I(s) is the gray-level of pixel s, $\sigma_{k}$ is the gray-level variance of the region $R_{k}$ and $A_{k}$ its surface.

Complementary to *NU*, *RC* is an inter-region metric that measures the gray-level contrast between regions to evaluate the dissimilarity of areas in the segmentation result. Both metrics are high to indicate good segmentation.

(22) $$RC=\sum_{i=1}^{N_{R}}\sum_{j=1}^{N_{R}}\frac{l_{ij}}{l_{i}}\frac{\left|m_{i}-m_{j}\right|}{m_{i}+m_{j}}\times \frac{1}{\sum_{i=1}^{N_{R}}w_{i}}$$

where $N_{R}$ represents the number of regions, $m_{i}$ is the average gray-level of the region $R_{i}$, $w_{i}$ is a weight factor, $l_{i}$ is the perimeter length of the region $R_{i}$, and $l_{ij}$ represents the border length between the regions.

### 6.4. Experimental Result of Image Segmentation

**Figure 3:**
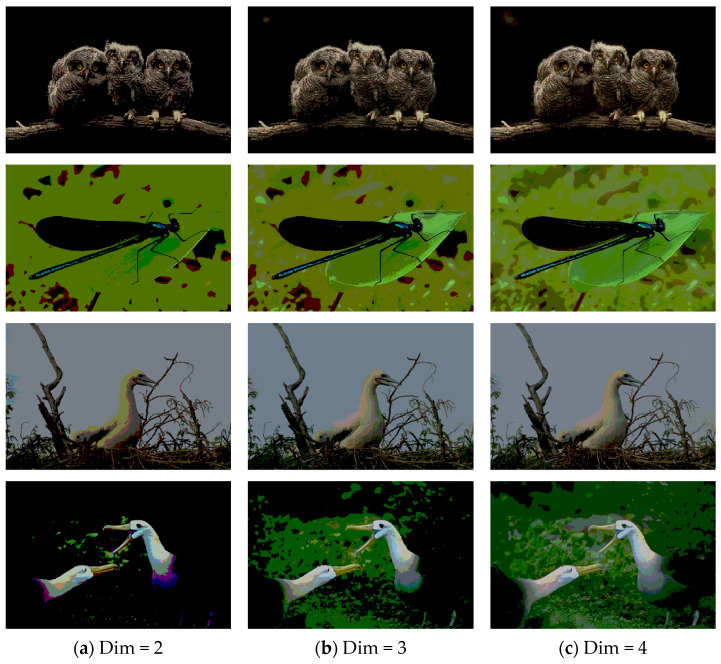
Diagram of segmented images by proposed method.

In this section, we present the segmented results of LDGTO compared with other peers. Several representative segmented results of the proposed algorithm are shown in Figure 3. It is obvious that the proposed method effectively separates the target from the background, and the quality of the segmentation result becomes superior with the threshold number increases. To quantitatively evaluate the quality of the segmented image, the NU and RC metrics are applied. The evaluation result for each algorithm is shown in Table 8 and Table 9. For the two metrics, the higher the value, the better the quality. We can observe from the table that our proposed LDGTO appraises excellent performance. To present a detailed description, the more specific analyses are provided as follows.

Firstly, NU calculates the homogeneity of the characteristics throughout the whole area by variances. It indicates better segmentation when the features over the same categories have better uniformity.

- Analyze the experimental results of P1-1 to P1-6 images in detail. According to the data in Table 9, the LDGTO algorithm performs very well in the segmentation effect of this group of images. In the P1-1 image, the NU value of LDGTO at 2 thresholds is 1.25 × 10^−1^, which is significantly better than other algorithms (e.g., the NU value of both MFO and POA is 9.10 × 10^−2^), showing that LDGTO can capture the features of the region better at low thresholds. In the subsequent 3- and 4-threshold conditions, LDGTO continues to maintain its dominance, especially in the 4-threshold condition, where the NU value of LDGTO is 1.53 × 10^−1^, which exceeds that of MFO (1.22 × 10^−1^) and POA (1.21 × 10^−1^). This result shows that with the increase of threshold, the segmentation effect of LDGTO does not decrease but further improves and can better separate the details in the region;
- The P2-1 to P2-5 images are analyzed. LDGTO also performs very well in this set of images, especially in the P2-1 and P2-5 images. The NU values of LDGTO are 2.06 × 10^−1^ and 1.12 × 10^−1^ under 2 thresholds, respectively, which are both better than other algorithms. In these two images, LDGTO not only performs well under lower thresholds but also maintains high segmentation quality under 3 thresholds, especially in the P2-4 image. The NU value of LDGTO is 1.48 × 10^−1^, which is significantly better than other algorithms. This indicates that LDGTO not only performs well on simple images but also can effectively handle more complex images.

Then, RC evaluates the dissimilarity of regions in the segmentation result. The RC index values of the segmented results achieved by algorithms are shown in Table 10.

- The LDGTO algorithm significantly outperforms the other algorithms in the P1 series of images, especially in 2D and 3D dimensions. Overall, LDGTO presents low RC values in most cases, showing its strong optimization ability and stability. Especially in more complex images such as P1-1, P1-2, and P1-3, the RC values of LDGTO are significantly lower than those of other algorithms, which suggests that LDGTO can better adapt to image segmentation tasks, especially in higher dimensions, and still maintains a low RC value. In 2D dimensions, LDGTO often shows optimal performance, while with the increase of dimensions (3D, 4D), the performance of some other algorithms, such as MFO, POA, and PSO, starts to approach LDGTO gradually, especially in the 4D dimensions of P1-4, P1-5, and P1-6, the RC value of LDGTO can still maintain a more stable advantage;
- The RC performance of the P2 series images is relatively different from the P1 series, with the overall performance leveling off. In several images of the P2 series, LDGTO still leads the way, especially in the 2D and 3D dimensions, where LDGTO has lower RC values than other algorithms. Specifically, in the P2-1 image, LDGTO has superior RC values in all dimensions (2D, 3D, and 4D), especially in the 4D dimension, where LDGTO has an RC value of 6.28 × 10^−1^, which is significantly lower than other algorithms. However, the performance of the algorithms in the P2 series is relatively close as the dimensionality increases. In the P2-4 and P2-5 images, although the RC value of LDGTO is still lower in the 2D dimension, the difference between the algorithms narrows in higher dimensions. For example, in the 3D dimension of P2-4, the RC value of LDGTO is 1.00 × 10^−1^, which performs relatively close to the other algorithms, showing the relative balance of the algorithms in some cases. Overall, the RC values of the P2 series of images perform relatively close to each other, but LDGTO still maintains the optimal performance in most cases, especially in high dimensions, proving the robustness of LDGTO in processing complex images.

Taken together, the LDGTO algorithm outperforms most of the compared algorithms in the P1-1 to P1-6 and P2-1 to P2-5 images, especially in terms of the stability of segmentation effect and feature separation ability. LDGTO shows a clear advantage. In the 11 test images, LDGTO has leading NU values in most cases, showing its efficiency and accuracy in image segmentation tasks. Therefore, it can be concluded that LDGTO is an algorithm with high robustness and excellent performance in different types of image segmentation tasks, which is worth promoting and using in practical applications.

## 7. LDGTO Handles Four-Bar Transplanter Mechanism Design

Transplanting mechanization can overcome the limitations of traditional manual work with high labor intensity and low efficiency and effectively improve the yield and quality of crops. The four-bar mechanism can realize various complex functions such as moving, swinging and rotating according to the preset motion law, which is characterized by high motion accuracy, long travel, and low wear. In this section, the optimal design problem of the four-bar mechanism of the planting machine is investigated, the planar four-bar mechanism is used to generate the ideal motion trajectory, and the size of the mechanism is inverted by the desired trajectory. In practice, the dimensional parameters of the member determine the motion law of the four-bar mechanism, which is its main source of error. Due to the complex nonlinear relationship between the motion law of the mechanism and the parameters of the member, this makes the optimal design of the mechanism a challenging subject.

### 7.1. Problem Description

**Figure 4:**
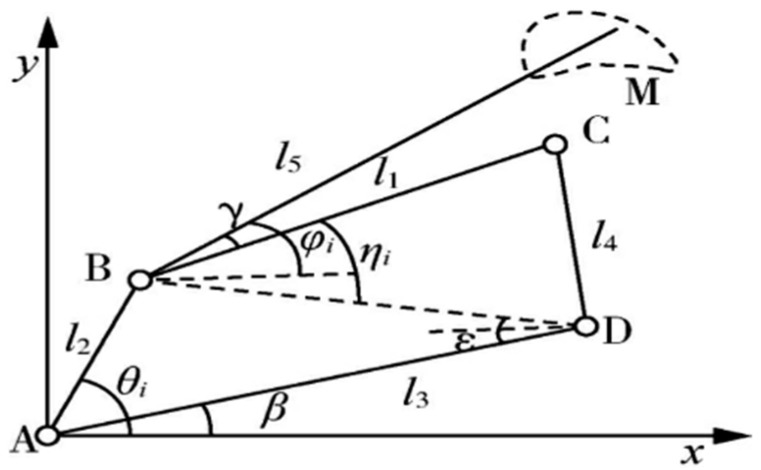
Schematic diagram of four-bar transplanter.

As shown in Figure 4, the simplified diagram of the four-rod mechanism of the planting machine, ABCD is a planar 2-degree-of-freedom four-rod, rod AB is a servomotor-driven drive rod, rod BC is a linkage, rod CD is a rocker, and rod AD is a frame. In this paper, the origin of the four-rod planting mechanism is chosen as point A, a right-angle coordinate system is established, and 8 discrete points are taken according to the preset ideal trajectory.

It is required that when rod AB rotates one week, point M on the connecting rod BC completes the trajectory on the figure, and the discrete point positions are shown in Table 11. Where *i* is the discrete point number, *S_xi_* and *S_yi_* are the *x*-axis and *y*-axis coordinates of the ideal trajectory, respectively.

### 7.2. Problem Modeling

From the geometric relationship shown in Figure 1, it can be seen that when the position of point A is fixed, the trajectory of the point M on the connecting rod BC depends mainly on the length *l*_1–5_ of each connecting rod and the angular parameter $\beta ,\gamma ,\theta_{0}$, where $\theta_{0}$ is the initial angle between AB and AD, so the design variable is:

(23) $$X=[x_{1},x_{2},x_{3},x_{4},x_{5},\beta ,\gamma ,\theta_{0}]$$

From the geometric relationship in the figure:

(24) $$\left\{\begin{array}{l} M_{xi}=l_{2}\cos\theta_{i}+l_{5}\cos\phi_{i}=x_{2}\cos\theta_{i}+x_{5}\cos\phi_{i}\\ M_{yi}=l_{2}\sin\theta_{i}+l_{5}\sin\phi_{i}=x_{2}\sin\theta_{i}+x_{5}\sin\phi_{i}\\ \theta_{i}=\theta_{0}+\beta +\Delta \theta_{i}\\ \phi_{i}=x_{7}+\eta_{i}-(\varepsilon_{i}-x_{6})\\ \varepsilon_{i}=\arctan\frac{x_{2}\sin(\theta_{i}-x_{6})}{x_{1}-x_{2}\cos(\theta_{i}-x_{6})}\\ \eta_{i}=\arccos\frac{{x_{1}}^{2}+{x_{2}}^{2}+{x_{3}}^{2}-{x_{4}}^{2}-2x_{1}x_{2}\cos(\theta_{i}-x_{6})}{2x_{3}\sqrt{{x_{1}}^{2}+{x_{2}}^{2}-2x_{1}x_{2}\cos(\theta_{i}-x_{6})}} \end{array}\right.$$

The goal of the optimization design is to make the motion trajectory of point *M* conform to the preset trajectory as much as possible, which can be understood as the minimization of the positional deviation with respect to the 8 discrete points, so the objective function is established in accordance with the root-mean-square of the error between the coordinates of the point M and the coordinates of the discrete points (*M_xi_*, *M_yi_*), i.e.,:

(25) $$f(X)=\sqrt{\frac{\sum_{i=1}^{8}\left[{\left(M_{xi}-S_{xi}\right)}^{2}+{\left(M_{yi}-S_{yi}\right)}^{2}\right]}{8}}$$

Six constraints imposed by the crank existence condition:

(26) $$\left\{\begin{array}{l} l_{2}\leq l_{i}(\;i=1,\;3,\;4)\Rightarrow g_{1,2,3}(X)=x_{2}-x_{i}\leq 0\\ l_{2}+l_{1}\leq l_{3}+l_{4}\Rightarrow g_{4}(X)=x_{2}+x_{1}-x_{3}-x_{4}\leq 0\\ l_{2}+l_{3}\leq l_{1}+l_{4}\Rightarrow g_{5}(X)=-x_{1}+x_{2}+x_{3}-x_{4}\leq 0\\ l_{2}+l_{4}\leq l_{1}+l_{3}\Rightarrow g_{6}(X)=-x_{1}+x_{2}-x_{3}+x_{4}\leq 0 \end{array}\right.$$

One constraint imposed by the minimum transmission angle condition:

(27) $$\left\{\begin{array}{l} \cos\delta_{\min}\frac{x_{3}^{2}+x_{4}^{2}-{\left(x_{1}-x_{2}\right)}^{2}}{2x_{3}x_{4}}\leq \cos30^{\circ }\\ g_{7}(X)=x_{3}^{2}+x_{4}^{2}-{\left(x_{1}-x_{2}\right)}^{2}-2x_{3}x_{4}\cos30^{\circ }\leq 0 \end{array}\right.$$

Thirteen constraints imposed by design variable boundary conditions:

(28) $$\left\{\begin{array}{l} l_{1}\leq 60\Rightarrow g_{8}(X)=x_{1}-60\leq 0\\ 8\leq l_{2}\leq 30\Rightarrow g_{9}(X)=8-x_{2}\leq 0\\ g_{10}(X)=x_{2}-30\leq 0\\ l_{3}\leq 60\Rightarrow g_{11}(X)=x_{3}-60\leq 0\\ l_{4}\leq 40\Rightarrow g_{12}(X)=x_{4}-40\leq 0\\ 10\leq l_{5}\leq 30\Rightarrow g_{13}(X)=10-x_{5}\leq 0\\ \;\;\;\;\;\;\;\;\;\;\;\;\;\;g_{14}(X)=x_{5}-30\leq 0\\ -20^{\circ }\leq \beta \leq 30^{\circ }\Rightarrow g_{15}(X)=-x_{6}-20\leq 0\\ \;\;\;\;\;\;\;\;\;\;\;\;\;\;\;\;\;\;g_{16}(X)=x_{6}-30\leq 0\\ -0^{\circ }\leq \gamma \leq 15^{\circ }\Rightarrow g_{17}(X)=0-x_{7}\leq 0\\ \;\;\;\;\;\;\;\;\;\;\;\;\;\;\;\;\;g_{18}(X)=x_{7}-15\leq 0\\ 0^{\circ }\leq \theta_{0}\leq 60^{\circ }\Rightarrow g_{19}(X)=0-x_{8}\leq 0\\ \;\;\;\;\;\;\;\;\;\;\;\;\;\;\;\;\;g_{20}(X)=x_{8}-60\leq 0 \end{array}\right.$$

In summary, the mathematical model for this optimization design is:

(29) $$\begin{array}{l} \min f(X)\\ s.t.\;g_{i}\leq 0\;(i=1,2...20) \end{array}$$

### 7.3. Experimental Result of Mechanism Design

LDGTO is applied to optimize the design of the model of the four-bar mechanism of the above planting machine, and the optimal dimensional parameters of each variable finally obtained by the algorithm of this paper under the given constraints are:

(30) $$X^{*}=\left[\begin{array}{l} x_{1}^{*}\\ x_{2}^{*}\\ x_{2}^{*}\\ x_{4}^{*}\\ x_{5}^{*}\\ x_{6}^{*}\\ x_{7}^{*}\\ x_{8}^{*} \end{array}\right]=\left[\begin{array}{l} 56.90\\ 9.06\\ 46.11\\ 27.68\\ 23.40\\ 17.92\\ 0.96\\ 43.83 \end{array}\right]$$

The value of its objective function is. In order to make a fair comparison, under the fixed mathematical model, compared with the results of the iterative solution by genetic algorithm tool in MATLAB, it is obvious that the design parameters optimized by the method of this paper have a smaller value of the objective function, which means that the dimensional parameters of the components of the four-bar mechanism of the planting machine designed by the method of this paper are more reasonable, and the overall mechanism has a smaller deviation of the trajectory.

## 8. Conclusions

Labor division and self-organization are fundamental mechanisms in biological systems for achieving efficient collective intelligence, yet these principles are overlooked in conventional Gorilla Troops Optimizer (GTO) algorithms, limiting their adaptability in many optimization scenarios. To address this gap, this study proposes LDGTO, an enhanced GTO variant that integrates natural labor division mechanisms into swarm intelligence. The algorithm introduces a dual-phase framework: a task allocation phase employing a stimulus-response model to dynamically assign exploration and exploitation tasks based on environmental feedback, and an outcome allocation phase implementing three behavioral modes (self-enhancement, competence maintenance, and elimination) aligned with developmental stages of individuals (elite, average, and underperforming). This design enables adaptive role plasticity, allowing gorillas to adjust strategies autonomously in response to search dynamics. Extensive evaluations on three sets of benchmark functions (12 unimodal, 25 multimodal, and 10 combinatorial) demonstrate LDGTO’s superior performance compared to three GTO variants and seven state-of-the-art competitors. Real-world applications in four-bar transplanter mechanism design and color image segmentation further validate its practicality and robustness. These advancements provide an effective tool for engineers to tackle complex optimization problems in mechanical design, pattern recognition, and resource allocation while offering researchers a methodological framework for enhancing bio-inspired algorithms.

Future work will explore LDGTO’s applicability to multi-objective optimization, investigate its scalability for ultra-large-scale problems, and refine labor division models through interdisciplinary collaboration with biologists. This study bridges biological principles with computational optimization, paving the way for innovative solutions in swarm intelligence and industrial applications.

## Figures and Tables

**Table 1:** Characteristics of multimodal test functions.

Num	Function Name	Range	Dim	Optimum
MF1	Schwefel’s 2.26	[−500, 500]	50	0
MF2	Rastrigin	[−5.12, 5.12]	50	0
MF3	Periodic	[−10, 10]	50	0.9
MF4	Qing	[−500, 500]	50	0
MF5	Alpine N.1	[−10, 10]	50	0
MF6	Xin-She Yang	[−5, 5]	50	0
MF7	Ackley	[−32, 32]	50	0
MF8	Trignometric 2	[−500, 500]	50	1
MF9	Salomon	[−100, 100]	50	0
MF10	Styblinski-Tang	[−5, 5]	50	−1958.299
MF11	Griewank	[−100, 100]	50	0
MF12	Xin-She Yang 4	[−10, 10]	50	−1
MF13	Xin-She Yang 2	[−2 pi, 2 pi]	50	0
MF14	Gen. Penalized	[−50, 50]	50	0
MF15	Penalized	[−50, 50]	50	0
MF16	Egg crate	[−5, 5]	2	0
MF17	Ackley N.3	[−32, 32]	2	−195.629
MF18	Adjiman	[−1, 2]	2	−2.02181
MF19	Bird	[−2 pi, 2 pi]	2	−106.7645
MF20	Camel Six Hump	[−5, 5]	2	−1.0316
MF21	Branin RCOS	[−5, 10]	2	0.3978873
MF22	Hartman 3	[0, 1]	3	−3.862782
MF23	Hartman 6	[0, 1]	6	−3.32237
MF24	Cross-in-tray	[−10, 10]	2	−2.0626121
MF25	Bartels Conn	[−500, 500]	2	1

**Table 2:** Characteristics of unimodal test functions.

Num	Function Name	Range	Dim	Optimum
UF1	Sphere	[−100, 100]	50	0
UF2	Quartic Noise	[−1.28, 1.28]	50	0
UF3	Powell Sum	[−1, 1]	50	0
UF4	Schwefel’s 2.20	[−100, 100]	50	0
UF5	Schwefel’s 2.21	[−100, 100]	50	0
UF6	Step	[−100, 100]	50	0
UF7	Stepint	[−5.12, 5.12]	50	0
UF8	Dixon and Price	[−10, 10]	50	0
UF9	Powell Singular	[−4, 5]	50	0
UF10	Perm 0, D, Beta	[−5, 5]	5	0
UF11	Three-Hump Camel	[−5, 5]	2	0
UF12	Schaffer N.4	[−100, 100]	2	0.29257

**Table 3:** Unimodal function optimization results.

Num	GTOT	mGTO	GTO	LDGTO
UF1	1.60 × 10^4^	6.67 × 10^4^	−2.02 × 10^4^	3.25 × 10^−74^
UF2	6.23 × 10^1^	2.82 × 10^2^	−2.17 × 10^2^	1.47 × 10^−1^
UF3	3.94 × 10^−1^	5.87 × 10^−1^	−1.51 × 10^−1^	1.53 × 10^−1^
UF4	7.31 × 10^2^	1.08 × 10^3^	−3.29 × 10^2^	1.88 × 10^−59^
UF5	4.44 × 10^1^	4.73 × 10^1^	5.19 × 10^1^	4.38 × 10^−72^
UF6	1.43 × 10^4^	1.10 × 10^5^	−8.45 × 10^4^	3.89 × 10^−1^
UF7	−2.75 × 10^2^	−2.75 × 10^2^	−2.75 × 10^2^	−2.77 × 10^2^
UF8	1.86 × 10^6^	2.78 × 10^6^	−2.65 × 10^6^	9.38 × 10^−1^
UF9	2.86 × 10^3^	6.87 × 10^3^	−6.28 × 10^3^	1.96 × 10^−5^
UF10	−4.71 × 10^2^	6.20 × 10^2^	1.23 × 10^3^	3.55 × 10^1^
UF11	−6.87 × 10^−48^	−4.06 × 10^−47^	2.34 × 10^−46^	1.43 × 10^−63^
UF12	2.93 × 10^−1^	2.93 × 10^−1^	2.93 × 10^−1^	3.01 × 10^−1^
+/=/−	10/0/2	10/0/2	9/0/3	-

**Table 4:** Multimodal function optimization results.

Num	GTOT	mGTO	GTO	LDGTO
MF1	1.43 × 10^2^	1.47 × 10^2^	1.76 × 10^2^	2.10 × 10^0^
MF2	3.04 × 10^2^	2.59 × 10^2^	4.07 × 10^2^	5.55 × 10^−4^
MF3	3.65 × 10^0^	4.11 × 10^0^	−6.38 × 10^−1^	3.40 × 10^−1^
MF4	3.41 × 10^10^	2.63 × 10^11^	−5.57 × 10^11^	1.23 × 10^4^
MF5	2.70 × 10^1^	6.90 × 10^1^	5.50 × 10^1^	1.60 × 10^−4^
MF6	−6.63 × 10^12^	−1.06 × 10^014^	9.09 × 10^13^	7.48 × 10^−13^
MF7	1.10 × 10^1^	1.81 × 10^1^	−1.23 × 10^1^	1.96 × 10^−10^
MF8	1.99 × 10^6^	2.32 × 10^6^	−1.25 × 10^7^	1.96 × 10^−10^
MF9	1.37 × 10^1^	2.90 × 10^1^	−1.04 × 10^2^	5.96 × 10^−50^
MF10	−1.46 × 10^3^	−1.87 × 10^3^	−1.10 × 10^3^	−1.96 × 10^3^
MF11	1.42 × 10^1^	2.41 × 10^1^	−1.13 × 10^2^	0.00 × 10^0^
MF12	−3.47 × 10^−1^	−1.00 × 10^0^	1.39 × 10^0^	−1.00× 10^0^
MF13	6.25 × 10^−21^	−1.39 × 10^−20^	2.94 × 10^−20^	1.70× 10^−108^
MF14	2.04 × 10^8^	1.41 × 10^9^	−1.10 × 10^9^	1.70× 10^−108^
MF15	3.55 × 10^8^	3.81 × 10^8^	−1.92 × 10^9^	1.07 × 10^−2^
MF16	−8.34 × 10^−80^	−1.51 × 10^−79^	1.23 × 10^−78^	6.91 × 10^−44^
MF17	−1.96 × 10^2^	−1.96 × 10^2^	−1.96 × 10^2^	−1.96 × 10^2^
MF18	−2.02 × 10^0^	−2.02 × 10^0^	−2.02 × 10^0^	−2.02 × 10^0^
MF19	−1.07 × 10^2^	−1.07 × 10^2^	−1.07 × 10^2^	−1.07 × 10^2^
MF20	−1.03 × 10^0^	−1.03 × 10^0^	−1.03 × 10^0^	−1.03 × 10^0^
MF21	3.99 × 10^−1^	4.00 × 10^−1^	3.90 × 10^−1^	3.98 × 10^−1^
MF22	−3.86 × 10^0^	−3.83 × 10^0^	−3.91 × 10^0^	−3.86 × 10^0^
MF23	−3.14 × 10^0^	−3.06 × 10^0^	−3.56 × 10^0^	−3.32 × 10^0^
MF24	−2.06 × 10^0^	−2.06 × 10^0^	−2.06 × 10^0^	−2.06 × 10^0^
MF25	3.52 × 10^1^	6.84 × 10^1^	−6.37 × 10^1^	5.60 × 10^−1^
+/=/−	20/4/1	19/5/1	20/4/1	-

**Table 5:** Combined function optimization results.

Functions		PSO	MFO	SCA	GPC	LDGTO
CF1	Mean	1.16 × 10^3^	1.16 × 10^3^	1.14 × 10^3^	1.13 × 10^3^	2.05 × 10^3^
	Std	1.22 × 10^3^	1.22 × 10^3^	1.20 × 10^3^	1.20 × 10^3^	5.69 × 10^0^
CF2	Mean	1.15 × 10^3^	1.15 × 10^3^	1.20 × 10^3^	1.22 × 10^3^	2.29 × 10^3^
	Std	1.21 × 10^3^	1.21 × 10^3^	1.26 × 10^3^	1.28 × 10^3^	4.15 × 10^0^
CF3	Mean	1.31 × 10^3^	1.32 × 10^3^	1.33 × 10^3^	1.33 × 10^3^	2.45 × 10^3^
	Std	1.38 × 10^3^	1.39 × 10^3^	1.40 × 10^3^	1.40 × 10^3^	2.38 × 10^1^
CF4	Mean	1.37 × 10^3^	1.38 × 10^3^	1.40 × 10^3^	1.40 × 10^3^	2.53 × 10^3^
	Std	1.45 × 10^3^	1.46 × 10^3^	1.47 × 10^3^	1.48 × 10^3^	1.13 × 10^2^
CF5	Mean	1.45 × 10^3^	1.48 × 10^3^	1.49 × 10^3^	1.49 × 10^3^	2.80 × 10^3^
	Std	1.53 × 10^3^	1.56 × 10^3^	1.57 × 10^3^	1.57 × 10^3^	1.10 × 10^2^
CF6	Mean	1.67 × 10^3^	1.49 × 10^3^	1.57 × 10^3^	1.57 × 10^3^	2.85 × 10^3^
	Std	1.80 × 10^3^	1.57 × 10^3^	1.65 × 10^3^	1.66 × 10^3^	1.32 × 10^2^
CF7	Mean	1.55 × 10^3^	1.55 × 10^3^	1.55 × 10^3^	1.55 × 10^3^	2.98 × 10^3^
	Std	1.63 × 10^3^	1.63 × 10^3^	1.64 × 10^3^	1.64 × 10^3^	1.44 × 10^1^
CF8	Mean	1.68 × 10^3^	1.66 × 10^3^	1.65 × 10^3^	1.68 × 10^3^	3.23 × 10^3^
	Std	1.77 × 10^3^	1.75 × 10^3^	1.74 × 10^3^	1.77 × 10^3^	1.16 × 10^2^
CF9	Mean	1.62 × 10^3^	1.61 × 10^3^	1.62 × 10^3^	1.62 × 10^3^	3.11 × 10^3^
	Std	1.71 × 10^3^	1.69 × 10^3^	1.71 × 10^3^	1.71 × 10^3^	5.15 × 10^1^
CF10	Mean	4.56 × 10^5^	5.21 × 10^5^	9.25 × 10^5^	6.34 × 10^5^	1.40 × 10^5^
	Std	9.68 × 10^5^	7.63 × 10^5^	1.11 × 10^6^	6.99 × 10^5^	1.20 × 10^5^
+/=/−		10/0/0	10/0/0	10/0/0	10/0/0	**-**

**Table 6:** Comparison with DIRECT.

Functions	Value		Time(s)	
	LDGTO	DIRECT	LDGTO	DIRECT
CF1	2.05 × 10^3^	2.00 × 10^3^	10.62	115.27
CF2	2.29 × 10^3^	2.10 × 10^3^	11.57	192.45
CF3	2.45 × 10^3^	2.39 × 10^3^	9.84	92.66
CF4	2.53 × 10^3^	2.42 × 10^3^	12.36	106.91
CF5	2.80 × 10^3^	2.80 × 10^3^	14.47	169.41
CF6	2.85 × 10^3^	2.15 × 10^3^	12.93	114.57
CF7	2.98 × 10^3^	2.98 × 10^3^	11.72	120.63
CF8	3.23 × 10^3^	3.23 × 10^3^	10.26	147.85
CF9	3.11 × 10^3^	2.56 × 10^3^	11.56	123.58
CF10	1.40 × 10^5^	1.40 × 10^5^	16.54	220.09
+/=/−		2/4/4		10/0/0

**Table 7:** Analysis of parameter *σ* in LDGTO.

Functions	$\sigma =10^{-6}$	$\sigma =10^{-5}$	$\sigma =10^{-4}$	$\sigma =10^{-3}$	$\sigma =10^{-2}$
CF1	2.26 × 10^3^	2.02 × 10^3^	2.05 × 10^3^	2.35 × 10^3^	2.61 × 10^3^
CF2	2.29 × 10^3^	2.00 × 10^3^	2.29 × 10^3^	2.29 × 10^3^	2.45 × 10^3^
CF3	2.51 × 10^3^	2.45 × 10^3^	2.45 × 10^3^	2.45 × 10^3^	2.56 × 10^3^
CF4	2.53 × 10^3^	2.06 × 10^3^	2.53 × 10^3^	2.57 × 10^3^	2.61 × 10^3^
CF5	2.95 × 10^3^	2.80 × 10^3^	2.80 × 10^3^	3.10 × 10^3^	2.05 × 10^3^
CF6	3.01 × 10^3^	2.85 × 10^3^	2.85 × 10^3^	2.67 × 10^3^	2.85 × 10^3^
CF7	2.73 × 10^3^	3.12 × 10^3^	2.98 × 10^3^	2.98 × 10^3^	2.98 × 10^3^
CF8	3.23 × 10^3^	3.65 × 10^3^	3.23 × 10^3^	3.23 × 10^3^	2.57 × 10^3^
CF9	3.11 × 10^3^	2.92 × 10^3^	3.11 × 10^3^	1.06 × 10^3^	2.43 × 10^3^
CF10	1.40 × 10^5^	1.52 × 10^4^	1.40 × 10^5^	1.68 × 10^4^	1.55 × 10^4^
+/=/−	6/3/1	7/3/1	-	6/4/1	7/2/1

**Table 8:** Benchmark images and their corresponding histograms.

Original Image					Histogram					Original Image					Histogram				
Animal																			
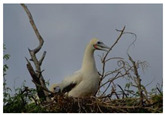					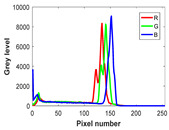					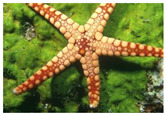					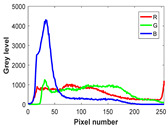				
(a) P1-1										(b) P1-2									
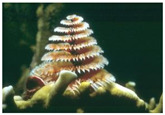					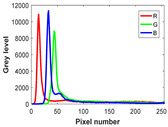					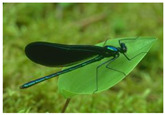					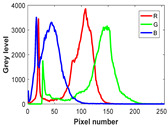				
(c) P1-3										(d) P1-4									
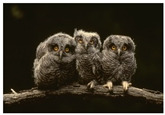					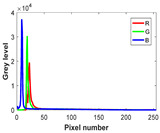					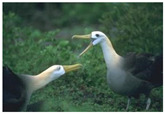					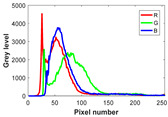				
(e) P1-5										(f) P1-6									
Human																			
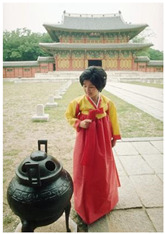				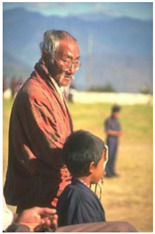				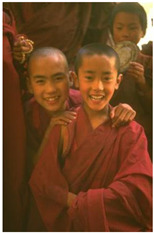				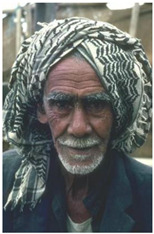				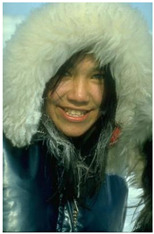			
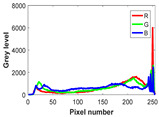				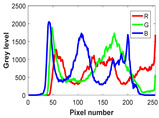				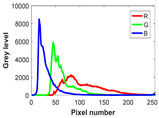				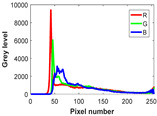				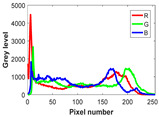			
(g) P2-1				(h) P2-2				(i) P2-3				(j) P2-4				(k) P2-5			

**Table 9:** NU performances of algorithms.

Image	Dim	LDGTO	MFO	ALO	WOA	PSO	SCA	MVO	POA
P1-1	2	1.25 × 10^−1^	9.10 × 10^−2^	9.10 × 10^−2^	9.10 × 10^−2^	9.10 × 10^−2^	8.30 × 10^−2^	9.10 × 10^−2^	1.00 × 10^−1^
	3	1.35 × 10^−1^	1.12 × 10^−1^	1.12 × 10^−1^	1.20 × 10^−1^	1.11 × 10^−1^	1.20 × 10^−1^	1.12 × 10^−1^	1.02 × 10^−1^
	4	1.53 × 10^−1^	1.22 × 10^−1^	1.25 × 10^−1^	1.30 × 10^−1^	1.24 × 10^−1^	1.52 × 10^−1^	1.24 × 10^−1^	1.21 × 10^−1^
P1-2	2	1.35 × 10^−1^	1.08 × 10^−1^	1.08 × 10^−1^	1.08 × 10^−1^	1.08 × 10^−1^	1.08 × 10^−1^	1.08 × 10^−1^	1.10 × 10^−1^
	3	1.05 × 10^−1^	1.03 × 10^−1^	1.03 × 10^−1^	1.03 × 10^−1^	1.03 × 10^−1^	1.05 × 10^−1^	1.03 × 10^−1^	1.05 × 10^−1^
	4	1.53 × 10^−1^	1.01 × 10^−1^	1.03 × 10^−1^	1.05 × 10^−1^	1.02 × 10^−1^	9.70 × 10^−2^	1.02 × 10^−1^	1.15 × 10^−1^
P1-3	2	2.53 × 10^−1^	2.14 × 10^−1^	2.14 × 10^−1^	2.14 × 10^−1^	2.14 × 10^−1^	2.19 × 10^−1^	2.14 × 10^−1^	2.13 × 10^−1^
	3	1.72 × 10^−1^	1.62 × 10^−1^	1.62 × 10^−1^	1.61 × 10^−1^	1.62 × 10^−1^	1.62 × 10^−1^	1.61 × 10^−1^	1.43 × 10^−1^
	4	1.70 × 10^−1^	1.28 × 10^−1^	1.28 × 10^−1^	1.27 × 10^−1^	1.28 × 10^−1^	1.35 × 10^−1^	1.27 × 10^−1^	1.29 × 10^−1^
P1-4	2	6.73 × 10^−2^	4.10 × 10^−2^	4.10 × 10^−2^	4.10 × 10^−2^	5.30 × 10^−2^	4.00 × 10^−2^	4.10 × 10^−2^	3.80 × 10^−2^
	3	1.05 × 10^−1^	4.90 × 10^−2^	5.20 × 10^−2^	4.60 × 10^−2^	4.80 × 10^−2^	5.00 × 10^−2^	4.90 × 10^−2^	5.50 × 10^−2^
	4	7.70 × 10^−2^	5.30 × 10^−2^	4.90 × 10^−2^	6.00 × 10^−2^	5.30 × 10^−2^	6.30 × 10^−2^	5.30 × 10^−2^	7.70 × 10^−2^
P1-5	2	2.03 × 10^−1^	1.57 × 10^−1^	1.57 × 10^−1^	1.60 × 10^−1^	1.57 × 10^−1^	1.57 × 10^−1^	1.57 × 10^−1^	1.62 × 10^−1^
	3	2.62 × 10^−1^	2.19 × 10^−1^	2.19 × 10^−1^	2.26 × 10^−1^	2.19 × 10^−1^	2.09 × 10^−1^	2.19 × 10^−1^	2.31 × 10^−1^
	4	2.55 × 10^−1^	2.01 × 10^−1^	2.01 × 10^−1^	2.08 × 10^−1^	2.00 × 10^−1^	2.20 × 10^−1^	1.99 × 10^−1^	2.11 × 10^−1^
P1-6	2	8.41 × 10^−2^	8.10 × 10^−2^	8.10 × 10^−2^	8.10 × 10^−2^	8.10 × 10^−2^	8.00 × 10^−2^	8.10 × 10^−2^	8.00 × 10^−2^
	3	7.90 × 10^−2^	7.30 × 10^−2^	7.70 × 10^−2^	7.90 × 10^−2^	7.30 × 10^−2^	7.60 × 10^−2^	7.70 × 10^−2^	5.50 × 10^−2^
	4	7.64 × 10^−2^	5.10 × 10^−2^	4.90 × 10^−2^	5.40 × 10^−2^	5.10 × 10^−2^	4.80 × 10^−2^	5.10 × 10^−2^	5.10 × 10^−2^
P2-1	2	2.06 × 10^−1^	2.04 × 10^−1^	2.04 × 10^−1^	2.05 × 10^−1^	2.04 × 10^−1^	2.06 × 10^−1^	2.04 × 10^−1^	1.80 × 10^−1^
	3	1.74 × 10^−1^	1.84 × 10^−1^	1.84 × 10^−1^	1.84 × 10^−1^	1.84 × 10^−1^	1.57 × 10^−1^	1.84 × 10^−1^	1.30 × 10^−1^
	4	1.94 × 10^−1^	1.56 × 10^−1^	1.49 × 10^−1^	1.50 × 10^−1^	1.49 × 10^−1^	1.35 × 10^−1^	1.45 × 10^−1^	1.24 × 10^−1^
P2-2	2	9.03 × 10^−2^	8.20 × 10^−2^	8.20 × 10^−2^	8.20 × 10^−2^	8.20 × 10^−2^	8.10 × 10^−2^	8.20 × 10^−2^	8.10 × 10^−2^
	3	1.04 × 10^−1^	8.30 × 10^−2^	8.30 × 10^−2^	8.30 × 10^−2^	8.30 × 10^−2^	9.10 × 10^−2^	8.30 × 10^−2^	8.90 × 10^−2^
	4	1.16 × 10^−1^	9.60 × 10^−2^	9.40 × 10^−2^	9.20 × 10^−2^	9.00 × 10^−2^	1.16 × 10^−1^	9.40 × 10^−2^	9.50 × 10^−2^
P2-3	2	1.61 × 10^−1^	1.58 × 10^−1^	1.58 × 10^−1^	1.58 × 10^−1^	1.58 × 10^−1^	1.45 × 10^−1^	1.58 × 10^−1^	1.61 × 10^−1^
	3	1.06 × 10^−1^	9.60 × 10^−2^	9.40 × 10^−2^	9.40 × 10^−2^	9.40 × 10^−2^	9.00 × 10^−2^	9.40 × 10^−2^	8.70 × 10^−2^
	4	1.04 × 10^−1^	8.50 × 10^−2^	8.50 × 10^−2^	8.60 × 10^−2^	8.50 × 10^−2^	7.60 × 10^−2^	8.40 × 10^−2^	8.50 × 10^−2^
P2-4	2	1.17 × 10^−1^	1.16 × 10^−2^	1.16 × 10^−1^	1.16 × 10^−1^	1.16 × 10^−1^	1.17 × 10^−1^	1.16 × 10^−1^	1.14 × 10^−1^
	3	1.48 × 10^−1^	1.15 × 10^−1^	1.15 × 10^−1^	1.15 × 10^−1^	1.15 × 10^−1^	1.15 × 10^−1^	1.15 × 10^−1^	1.17 × 10^−1^
	4	1.36 × 10^−1^	1.21 × 10^−1^	1.21 × 10^−1^	1.21 × 10^−1^	1.21 × 10^−1^	1.18 × 10^−1^	1.21 × 10^−1^	1.17 × 10^−1^
P2-5	2	1.12 × 10^−1^	8.70 × 10^−2^	8.70 × 10^−2^	8.70 × 10^−2^	8.70 × 10^−2^	8.70 × 10^−2^	8.70 × 10^−2^	8.40 × 10^−2^
	3	1.19 × 10^−1^	8.10 × 10^−2^	8.10 × 10^−2^	7.8 × 10^−2^	8.10 × 10^−2^	7.90 × 10^−2^	8.10 × 10^−2^	8.60 × 10^−2^
	4	9.56 × 10^−2^	7.50 × 10^−2^	7.50 × 10^−2^	8.00 × 10^−2^	8.00 × 10^−2^	8.40 × 10^−2^	7.90 × 10^−2^	8.40 × 10^−2^
+/=/−		~	32/0/1	32/0/1	30/1/1	32/0/1	28/4/0	32/0/1	29/4/0

**Table 10:** RC performances of algorithms.

Image	Dim	LDGTO	MFO	ALO	WOA	PSO	SCA	MVO	POA
P1-1	2	3.27 × 10^−2^	2.50 × 10^−2^	2.50 × 10^−2^	2.50 × 10^−2^	2.50 × 10^−2^	2.50 × 10^−2^	2.50 × 10^−2^	2.70 × 10^−2^
	3	3.31 × 10^−2^	3.00 × 10^−2^	3.00 × 10^−2^	3.10 × 10^−2^	2.90 × 10^−2^	3.00 × 10^−2^	3.00 × 10^−2^	2.70 × 10^−2^
	4	4.37 × 10^−2^	3.60 × 10^−2^	3.70 × 10^−2^	3.50 × 10^−2^	3.60 × 10^−2^	4.00 × 10^−2^	3.60 × 10^−2^	3.70 × 10^−2^
P1-2	2	2.53 × 10^−1^	2.09 × 10^−1^	2.09 × 10^−1^	2.09 × 10^−1^	2.09 × 10^−1^	2.10 × 10^−1^	2.09 × 10^−1^	1.99 × 10^−1^
	3	3.23 × 10^−1^	3.19 × 10^−1^	3.19 × 10^−1^	3.20 × 10^−1^	3.19 × 10^−1^	3.23 × 10^−1^	3.19 × 10^−1^	3.01 × 10^−1^
	4	4.54 × 10^−1^	3.89 × 10^−1^	3.90 × 10^−1^	4.02 × 10^−1^	3.93 × 10^−1^	3.82 × 10^−1^	3.93 × 10^−1^	4.29 × 10^−1^
P1-3	2	1.79 × 10^−1^	1.58 × 10^−1^	1.58 × 10^−1^	1.58 × 10^−1^	1.58 × 10^−1^	1.38 × 10^−1^	1.58 × 10^−1^	1.58 × 10^−1^
	3	1.77 × 10^−1^	1.54 × 10^−1^	1.54 × 10^−1^	1.54 × 10^−1^	1.54 × 10^−1^	1.77 × 10^−1^	1.54 × 10^−1^	1.45 × 10^−1^
	4	2.01 × 10^−1^	1.97 × 10^−1^	1.97 × 10^−1^	1.92 × 10^−1^	1.97 × 10^−1^	1.83 × 10^−1^	1.94 × 10^−1^	1.99 × 10^−1^
P1-4	2	6.22 × 10^−2^	4.20 × 10^−2^	4.20 × 10^−2^	4.20 × 10^−2^	5.10 × 10^−2^	4.00 × 10^−2^	4.20 × 10^−2^	4.30 × 10^−2^
	3	7.06 × 10^−2^	5.60 × 10^−2^	5.50 × 10^−2^	6.30 × 10^−2^	7.80 × 10^−2^	5.80 × 10^−2^	5.60 × 10^−2^	6.30 × 10^−2^
	4	1.06 × 10^−1^	1.01 × 10^−1^	9.30 × 10^−2^	9.20 × 10^−2^	1.01 × 10^−1^	1.06 × 10^−1^	1.01 × 10^−1^	9.10 × 10^−2^
P1-5	2	7.35 × 10^−2^	5.10 × 10^−2^	5.10 × 10^−2^	5.20 × 10^−2^	5.10 × 10^−2^	5.10 × 10^−2^	5.10 × 10^−2^	5.50 × 10^−2^
	3	6.59 × 10^−2^	4.60 × 10^−2^	4.60 × 10^−2^	4.60 × 10^−2^	4.60 × 10^−2^	4.70 × 10^−2^	4.60 × 10^−2^	4.90 × 10^−2^
	4	5.73 × 10^−2^	4.30 × 10^−2^	4.00 × 10^−2^	4.10 × 10^−2^	4.00 × 10^−2^	4.20 × 10^−2^	3.90 × 10^−2^	3.80 × 10^−2^
P1-6	2	7.35 × 10^−2^	6.60 × 10^−2^	6.60 × 10^−2^	6.60 × 10^−2^	6.60 × 10^−2^	6.60 × 10^−2^	6.60 × 10^−2^	6.40 × 10^−2^
	3	7.60 × 10^−2^	7.60 × 10^−2^	7.20 × 10^−2^	7.60 × 10^−2^	7.60 × 10^−2^	7.00 × 10^−2^	7.20 × 10^−2^	7.00 × 10^−2^
	4	9.06 × 10^−2^	7.90 × 10^−2^	7.90 × 10^−2^	7.90 × 10^−2^	7.90 × 10^−2^	8.00 × 10^−2^	7.90 × 10^−2^	8.20 × 10^−2^
P2-1	2	3.69 × 10^−1^	3.56 × 10^−1^	3.56 × 10^−1^	3.55 × 10^−1^	3.56 × 10^−1^	3.69 × 10^−1^	3.56 × 10^−1^	3.47 × 10^−1^
	3	4.50 × 10^−1^	4.20 × 10^−1^	4.20 × 10^−1^	4.19 × 10^−1^	4.20 × 10^−1^	3.91 × 10^−1^	4.20 × 10^−1^	4.09 × 10^−1^
	4	6.28 × 10^−1^	5.61 × 10^−1^	5.48 × 10^−1^	5.46 × 10^−1^	5.48 × 10^−1^	5.56 × 10^−1^	5.47 × 10^−1^	4.39 × 10^−1^
P2-2	2	2.01 × 10^−1^	1.91 × 10^−1^	1.91 × 10^−1^	1.91 × 10^−1^	1.91 × 10^−1^	1.92 × 10^−1^	1.91 × 10^−1^	1.87 × 10^−1^
	3	2.39 × 10^−1^	2.10 × 10^−1^	2.10 × 10^−1^	2.10 × 10^−1^	2.10 × 10^−1^	2.15 × 10^−1^	2.10 × 10^−1^	2.23 × 10^−1^
	4	2.74 × 10^−1^	2.59 × 10^−1^	2.60 × 10^−1^	2.58 × 10^−1^	2.60 × 10^−1^	2.64 × 10^−1^	2.60 × 10^−1^	2.36 × 10^−1^
P2-3	2	1.76 × 10^−1^	1.57 × 10^−1^	1.57 × 10^−1^	1.57 × 10^−1^	1.57 × 10^−1^	1.54 × 10^−1^	1.57 × 10^−1^	1.58 × 10^−1^
	3	1.92 × 10^−1^	1.89 × 10^−1^	1.92 × 10^−1^	1.92 × 10^−1^	1.92 × 10^−1^	1.93 × 10^−1^	1.92 × 10^−1^	1.91 × 10^−1^
	4	2.23 × 10^−1^	2.06 × 10^−1^	2.06 × 10^−1^	2.05 × 10^−1^	2.06 × 10^−1^	1.99 × 10^−1^	1.54 × 10^−1^	1.98 × 10^−1^
P2-4	2	1.21 × 10^−1^	1.10 × 10^−1^	1.10 × 10^−1^	1.10 × 10^−1^	1.10 × 10^−1^	1.08 × 10^−1^	1.10 × 10^−1^	1.13 × 10^−1^
	3	1.00 × 10^−1^	9.00× 10^−2^	9.00 × 10^−2^	9.00 × 10^−2^	9.00 × 10^−2^	9.30 × 10^−2^	9.00 × 10^−2^	8.00 × 10^−2^
	4	1.27 × 10^−1^	9.50× 10^−2^	9.60 × 10^−2^	9.60 × 10^−2^	9.50 × 10^−2^	9.20 × 10^−2^	9.60 × 10^−2^	9.00 × 10^−2^
P2-5	2	1.16 × 10^−1^	9.50 × 10^−2^	9.50 × 10^−2^	9.50 × 10^−2^	9.50 × 10^−2^	9.70 × 10^−2^	9.50 × 10^−2^	9.60 × 10^−2^
	3	1.55 × 10^−1^	1.26 × 10^−1^	1.26 × 10^−1^	1.25 × 10^−1^	1.26 × 10^−1^	1.24 × 10^−1^	1.26 × 10^−1^	1.23 × 10^−1^
	4	2.08 × 10^−1^	1.99 × 10^−1^	1.99 × 10^−1^	1.70 × 10^−1^	1.71 × 10^−1^	1.80 × 10^−1^	1.70 × 10^−1^	1.75 × 10^−1^
+/=/−		~	31/1/1	32/0/1	31/1/1	30/1/2	27/6/0	32/0/1	32/1/0

**Table 11:** Ideal trajectory motion coordinates.

Parameters	Values for Each Stage							
A	1	2	3	4	5	6	7	8
BC	26	23	20	17	13	10	20	30
M	16	16	17	16	15	11	6	12
$l_{1},l_{2},l_{3},l_{4},l_{5}$	0	22	44	66	88	129	221	314

## Data Availability

The original contributions presented in this study are included in the article. Further inquiries can be directed to the first author. Figures in the Table 6 comes from the open data set BSDS500 (The Berkeley Segmentation Dataset and Benchmark).
